# Windblown sediment transport and loss in a desert–oasis ecotone in the Tarim Basin

**DOI:** 10.1038/s41598-017-04971-4

**Published:** 2017-08-10

**Authors:** Huawei Pi, Brenton Sharratt, Jiaqiang Lei

**Affiliations:** 1 State Key Laboratory of Desert and Oasis Ecology, Xinjiang Institute of Ecology and Geography, Chinese Academy of Sciences, Urumqi, Xinjiang 830011 China; 2 University of Chinese Academy of Sciences, Beijing, 100049 China; 3 Washington State University, Pullman, WA 99164 USA; 4 USDA-ARS, 215 Johnson Hall, Washington State University, Pullman, WA 99164 USA; 5 State Key Laboratory of Desert and Oasis Ecology, Xinjiang Institute of Ecology and Geography, Chinese Academy of Sciences, Urumqi, Xinjiang 830011 China

## Abstract

The Tarim Basin is regarded as one of the most highly erodible areas in China. Desert comprises 64% of the land use in the Basin, but the desert–oasis ecotone plays a prominent role in maintaining oasis ecological security and stability. Yet, little is known concerning the magnitude of windblown sediment transport in a desert-oasis ecotone. Therefore, aeolian sediment transport and loss was assessed from a desert-oasis experimental site located near Alaer City in the northwestern Tarim Basin. Sediment transport and factors governing transport were measured during three high wind events in 2012 and four events in 2013. Sediment transport was measured to a height of 10 m using passive aeolian airborne sediment samplers. The mass flux profile over the eroding surface was well represented by the power-law (R^2^ > 0.77). Sediment loss from the site ranged from 118 g m^−2^ for the 20–24Apr 2012 wind event to 2925 g m^−2^ for the 31Mar–11Apr 2012 event. Suspension accounted for 67.4 to 84.8% of sediment loss across all high wind events. Our results indicate the severity of wind erosion in a desert-oasis ecotone and thus encourage adoption of management practices that will enhance oasis ecological security.

## Introduction

The Tarim Basin is regarded as one of the most important dust source regions in China^1, 2^. A major source of dust is that caused by wind erosion^3^. Indeed, wind erosion is a severe problem in the Tarim Basin due to the climatic and geographical conditions in the region^4^. The Basin is surrounded by the Tian Shan mountain range to the north, Kunlun mountain range to the south, and Pamir mountain range to the west. These tall mountains (elevations are more than 7,000 m) typically intercept atmospheric moisture transported into the region by prevailing westerly winds and greatly influence the atmospheric circulation across the region. As a result, the Tarim Basin is characterized by a desert environment. Sixty-four percent of the Basin is covered by desert. Very little precipitation (annual precipitation ranges from 10 to 100 mm) in the region suppresses growth of plants and minimizes aggregation or crusting of sand or soil particles. Furthermore, the Tarim Basin is one of the most highly erodible areas in China^4^.

Wind erosion processes involve transport of soil particles by creep, saltation and suspension. Aggregates or particles <0.1 mm in diameter generally are suspended above the surface while aggregates or particles 0.1~2.0 mm in diameter creep and saltate along the surface^5^. The transport of aggregates or particles is also influenced by particle shape and density. The suspension component is derived from direct emission of fine particles on the surface or abrasion of coarser particles either airborne or on the surface. The saltation component, however, is derived from particles bouncing along the surface as well as abrasion or break down of coarser particles^6^. The latest scientific findings show that aerodynamic entrainment is very important for the emission of dust on the surface^7, 8^. Suspension is more important than creep and saltation for air quality because fine particles in suspension are emitted into the troposphere or lower stratosphere and thus can be carried around the world. For example, *Creamean et al*.^9^ showed that dust in suspension from the Taklimakan Desert in China travelled to the U.S. within 13 days.

Severe wind erosion threatens ecosystem stability and air quality as a result of removing the finer and most fertile particles from the soil surface into the atmosphere. Wind erosion is influenced by land cover type (e.g., desert, grassland, cultivated land, desert–oasis ecotone) due to differences in characteristics of vegetation or soil. Desert-oasis ecotones play a prominent role of ensuring oasis ecological security and maintaining oasis ecological stability due to its location between an oasis and the neighboring desert. Oasis evolution consists of two opposing processes of oasification and desertification. Severe wind erosion will accelerate desertification and will result in fertile land transforming into desert, thus reducing oasis stability and ecological security.

Many studies have been conducted to assess the broad range of climatic, soil, and management conditions that influence wind erosion. For example, *Van Donk et al*.^10^ quantified wind erosion in the Mojave Desert of California. *Zobeck and Van Pelt* ^11^ indicated that dust flux estimates above an agricultural field were sensitive to measurement height. *Sharratt et al*.^12^ measured soil loss that ranged from 43 to 2320 kg ha^−1^ from winter wheat–summer fallow fields in the Columbia Plateau. *Zheng et al*.^13^ measured soil loss ranging from 0 to 142 g m^−2^ in croplands of the Tarim Basin. Wind erosion has been measured under various land use types including playa lakes^14^, cropland^11^, shrubland^15^, forest^16^ and desert^17^.

Few scientists have documented creep, saltation, and suspension components of wind erosion. *Chepil* ^18^ reported that saltation accounted for the greatest proportion of mass transport of agricultural soils by wind. *Stout and Zobeck* ^19^ indicated that saltation was the dominate mode of transport below at a height of 0.1 m whereas suspension dominated flow above 0.2 m in eroding agricultural field. *Tchakerian* ^20^ indicated that saltation accounted for 80% while suspension accounted for 5% of the transport load in deserts. *Hagen et al*.^21^ reported saltation flux can exceed 500 kg m^−2^ while suspension flux ranged from 0.5 to 200 kg m^−2^ within 1.65 m of the surface of an eroding agricultural field. However, suspension can be the dominate mode of transport of wind erosion. For example, *Sharratt et al*.^12^ found that 94% of sediment in transport above an eroding loessial soils was by suspension whereas 2% and 4% of sediment in transport was by respectively creep and saltation in the Columbia Plateau.

*Pi et al*.^22^ simulated wind erosion processes using the Single-event Wind Erosion Evaluation Program (SWEEP) in a desert-oasis ecotone in the Tarim Basin. Although the SWEEP adequately simulated creep plus saltation but not suspension, their data did not account for significant sediment flux within the surface boundary layer above 2 m. Field observations of sediment flux are typically constrained to within 2 m of the surface since the majority of sediment is transported and the vertical distribution of mass flux is well defined within 2 m of the surface^23, 24^. We are unaware of other studies documenting sediment transport in a desert-oasis ecotone. Therefore, this study assesses sediment transport and loss within the surface boundary layer of a desert–oasis ecotone in the Tarim Basin.

## Materials and Methods

The experiment was conducted in spring 2012 and 2013, within a desert–oasis ecotone, about 10 km south of Alaer City (40°27′N, 81°19′E, elevation of 992 m) in Xinjiang Province, China. The desert–oasis ecotone acts as an interactive zone between Alaer oasis and Taklimakan Desert and is close to the confluence of the Aksu, Hotan, and Yarkand Rivers; these rivers join to form the Tarim River in the northwest part of the Tarim Basin. The soil type was aeolian sand with 0.01% clay, 0.4% silt, and 99.6% sand. The natural vegetation was sparsely distributed and consisted of Saltbush (Atriplex polycarpa L.) and Manaplant Alhagi (Alhagi sparsifolia L.). The average annual precipitation of this region is 53 mm and the average annual temperature is 11.3 °C. The region is mainly affected by NW winds.

**Figure 1:**
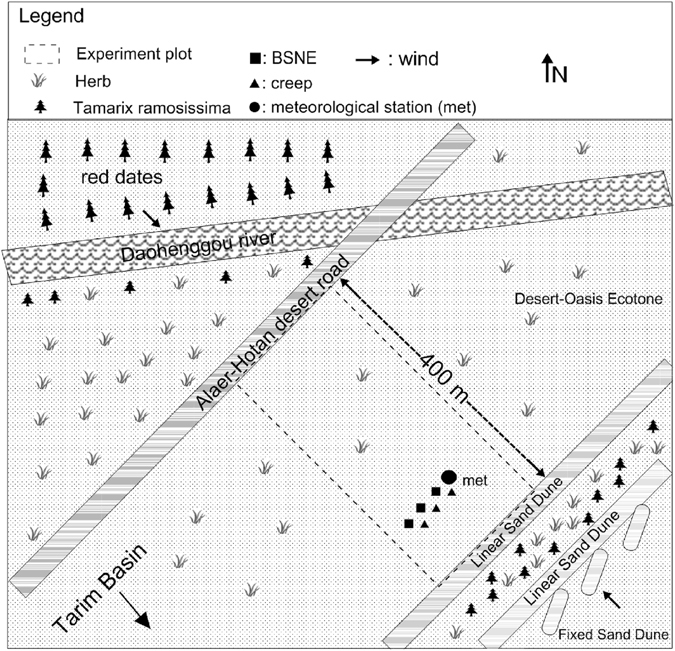
Location of instrumentation in the experimental plot with symbols representing placement of BSNE (◾) and creep (▴) collectors and meteorological (met) station (Generated by Adobe Photoshop 12.0, http://www.photoshop.com).

Sediment loss was assessed from a rectangle plot at the experimental site (Figure 1). An ephemeral tributary (Daohenggou River was 30 m wide and 1 m deep) and paved road (Alaer-Hotan Desert Road was 10 m wide and had 1 m of relief) provided “zero sediment flux boundaries” to minimize the influx of saltating particles to the plot under prevailing westerly winds. Fixed or stable dunes abutted the transverse dunes (2 m in height and 10 m wide) located on the southeast end of the plot. The transverse dune and intervening vegetation were assumed to provide a “zero sediment flux boundary” along the southeast border of the experimental plot when winds were from the southeast. A description of the experimental plot and “zero sediment flux boundaries” is given by *Pi et al*.^22^.

Two separate samplers were used to measure the total mass flux profile. Creep sediment collectors (also known as near-surface sampler) were used to obtain measurements within 0.025 m of the surface and BSNE (Big Spring Number Eight) airborne sediment collectors were used to obtain the mass flux at 0.1, 0.2, 0.5, 1.0, 1.5, 2.0, 3.6, 6.0, and 10.0 m above the surface. The creep samplers Custom Products, Big Springs, Texas) were placed at ground-level to measure sediment flux to a height of 0.025 m. Mass flux was assessed to a 10 m height since 10 m typically defines the height of the constant shear-stress layer or surface boundary layer^25^. Creep and BSNE collectors were positioned away from small shrub-coppice dunes and the sparse vegetation within the plot as much as possible to avoid obstructions to airflow caused by these dunes and vegetation. Sediment flux within 2 m of the surface was assessed at three locations spaced 3 m apart along a line perpendicular to the prevailing wind and near a meteorological tower. Sediment flux at 3.6, 6.0, and 10.0 m above the surface was assessed at one location (meteorological tower) in the plot. Previous studies showed that BSNE efficiencies change with particle size and wind speed^26^. The BSNE was the most recommended sampler for field measurements with an efficiency of 100% for sand having a geometric diameter similar to that at our field site (see below)^27^.

Sediment in the collectors was retrieved after each high wind event. Collectors were also examined weekly and cleared of debris if necessary. Sediment from the collectors was placed in plastic bags and transported to the laboratory where the sediment was air-dried, weighed, and sieved by hand through screens having 2, 0.84, and 0.1 mm openings to determine the aggregate or particle size distribution. For the purpose of this study, the 2–0.84, 0.84–0.1, and <0.1 mm size fractions obtained by sieving correspond to respective creep-size, saltation-size, and suspension-size particles^22^. We used a simple power-law expression to describe the vertical distribution of sediment captured by BSNE collectors^28^ which is

1 $${\rm{q}}={{\rm{\alpha }}{\rm{z}}}^{({\rm{-}}{\rm{\beta }})}$$

where q is sediment catch (kg m^2^), z is height (m) of the opening of the BSNE collector above the surface and α and β are fitted parameters. Eq. (1) was integrated from 0.025 to 10 m using the power equation to obtain total BSNE horizontal sediment flux (kg m^−1^) for the sample period. Total horizontal flux is the sum of the flux from both creep and BNSE collectors. Sediment loss was calculated as total horizontal flux divided by the distance between the collectors and “zero sediment flux boundary”.

Drift potential is the most important and frequently used parameter for assessing the effect of wind energy on sand flux^29^. This parameter represents the potential amount of sand drift^30^. In this study, we calculated *DP* for all observed sample periods so as to provide a glimpse of wind induced sediment flux. The drift *DP* was calculated using the equation^31^:

2 $$DP=\frac{{u}^{2}(u-{u\ast }_{{\rm{t}}})}{100}t$$

where DP is in vector units, *u* is the wind velocity (knots), u_*t_ is the threshold velocity (knots) and *t* is the amount of time the wind was above the threshold (%).

Wind direction was measured using a wind vane at a 10 m height and wind speed was measured using 3-cup anemometers at heights of 0.1, 0.5, 1, and 10 m above the surface. Micrometeorological sensors were monitored every 10 s and data recorded every 15 min by a datalogger (Model 10X, Campbell Scientific Inc., Logan, Utah).

## Results

Sediment was collected over three sampling periods in 2012 and four sampling periods in 2013. Each sampling period included at least one high wind event. A high wind event was initiated and terminated when the wind velocity exceeded the threshold wind velocity for 10 consecutive minutes^32^. The threshold velocity at height of 1 m was estimated as 4 m s^−1^ by *Pi et al*.^22^.

A summary of the wind characteristics for the seven sampling periods and the accompanying sediment loss are shown in Table 1. Sediment loss ranged from 118 g m^−2^ for the 20–24Apr 2012 sample period to 2925 g m^−2^ for the 31Mar–11Apr2012 sample period. For the latter period, the maximum wind speed was 8.3 m s^−1^ and winds were in excess of 4 m s^−1^ at a height of 1 m for 16 h.

**Table 1:** High wind event characteristics and total measured sediment loss partitioned by creep plus saltation, and suspension in the desert-oasis ecotone during 2012 and 2013.

Year	Sampling Period^a^	Events^b^	Hours^c^	Wind Dir^d^	Wind speed^e^		EL^f^	Total sediment loss	Creep	saltation	Suspension	Drift potential^g^ (*DP*)
					Mean	Max						
				degrees	m s^−1^		m	g m^−2^	g m^−2^	g m^−2^	g m^−2^	vector units
2012	31Mar–11Apr	5	16	161	5.3	8.3	111	2925	0	488	2437	124.1
	20–24Apr	3	7	277	4.9	5.7	381	118	0	18	100	13.5
	16–18May	2	8	297	4.8	6.6	315	634	0	108	526	60.7
2013	26–30Apr	3	8	155	4.4	5.6	106	153	0	50	103	6.8
	1–5May	3	20	63	4.6	6.6	324	213	0	58	155	33.3
	6–12May	3	13	124	5.3	7.2	102	467	0	80	387	49.7
	20–26May	6	30	140	5.2	6.9	100	389	0	90	299	32.8

^a^Dates over which eroded sediment was collected.

^b^Number of high wind events observed during the sampling period. A high wind event was defined by wind speeds that exceeded 4 m *s* ^−1^ at a height of 1 m.

^c^Number of hours during the sampling period for which wind speed exceeded 4 m *s* ^−1^ at a height of 1 m.

^d^Mean wind direction at 1 m height during the high wind events.

^e^Mean and maximum (max) wind speed at 1 m height during high wind events.

^f^EL is erosion length or distance from the sediment collectors to the non-erodible boundary in the direction of the prevailing wind.

^g^Drift potential (*DP*) in vector units for each observed sample periods.

The average wind speed for the 20–26May2013 period was 5.2 m/s, which was similar for the 31Mar–11Apr2012 period. The duration of winds in excess of 4 m s^−1^ for the 20–26May2013 sample period was twice that of the 31Mar–11Apr2012 sample period. Winds in excess of 4 m s^−1^ were sustained for 30 h for the 20–26May2013 sampling period and 16 h for the 31Mar–11Apr2012 period. Sediment loss for the 20–26May2013 sampling period, however, was 13% of the loss observed for the 31Mar–11Apr2012 period. This difference in sediment loss can be due to the longer duration of winds in excess of 6 m s^−1^ for the 31Mar–11Apr2012 sample period. For example, winds in excess of 6 m s^−1^ were sustained for 13 h during the 31 Mar–11Apr2012 sampling period and 5 h for the 20–26May2013 period^22^.

Drift potential (*DP*) may also explain the difference in sediment loss between 31Mar–11Apr2012 and 20–26May2013 sample periods. The calculated *DP* for the seven observed sample periods is presented in Table 1. The *DP* for the 31Mar–11Apr2012 sample period was nearly four times that of the 20–26May2013 period. Thus, this may in part explain the reason sediment loss for the 31Mar–11Apr2012 sample period was seven times greater than sediment loss for the 20–26May2013 period. We found a significant relationship between *DP* and measured sediment loss. Regression analysis between *DP* and measured sediment loss for the seven sample periods (y = 24.0x − 402.4, R² = 0.90) clearly indicate that wind is a dominant factor in wind erosion. Other factors such as biomass and random roughness may also influence wind erosion in this desert-oasis ecotone. For example, regression analysis between *DP* and sediment loss for the three sampling periods occurring in March and April indicated that 99% of the variation in sediment loss could be explained by the variation in DP (y = 24.4x − 109.0; R^2^ = 0.99). These sample periods occurred while vegetation was dormant. Regression analysis for the four sampling periods in May indicated that 81% of the variation in sediment loss could be explained by the variation in DP (y = 11.6x − 86.6; R² = 0.81). These sample periods occurred during development of vegetation at the experimental site. Thus, factors other than DP or wind influenced sediment loss during May. The correlation coefficient between maximum wind speed and sediment loss across the seven sampling periods was 0.70 and between mean wind speed and sediment loss was 0.28. Thus, maximum wind speed appeared to influence sediment loss more than mean wind speed.

## Discussion

Maximum sediment loss observed from an aeolian sand at our desert-oasis experimental site (2925 g m^−2^) was greater than the loss observed from a silt loam (60 g m^−2^)^10^ and clay loam in Colorado (150 g m^−2^)^33^, loam in Nebraska (210 g m^−2^)^33^, and loamy sand (2530 g m^−2^)^33^ and silt loam in Washington (232 and 1170 g m^−2^)^12, 33^. However, maximum sediment loss in this study was smaller than from a loamy sand in Texas (5620 g m^−2^)^33^.

The distance from the sediment collectors to the windward zero sediment flux or non-erodible plot boundary was determined by the wind direction. Wind direction was not always from the NW or SE during high wind events, so the distance between the collectors and non-erodible boundary varied among high wind events (Table 1). The shortest distance was 300 m when the wind direction was from the northwest and 100 m when the wind direction was from the southeast (Figure 1). *Stout* ^34^ indicated that measured mass flux at a fixed height will increase continually with fetch distance. However our results show that erosion under the longest fetch (381 m in Table 1) did not correspond to the maximum measured sediment loss.

**Figure 2:**
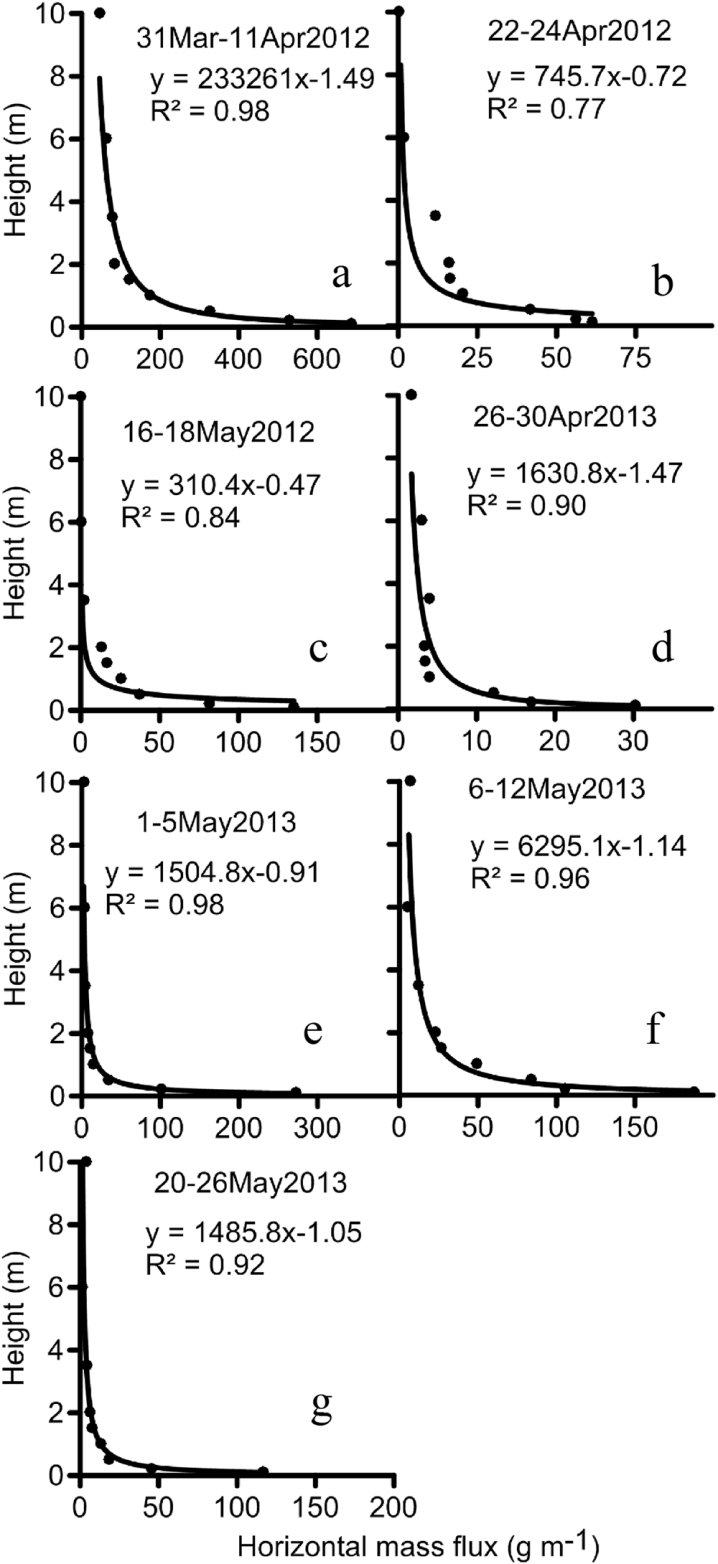
Horizontal mass flux as a function of height above the eroding surface during seven high wind events. Sediment was trapped by BSNE collectors positioned at nine heights (0.1–10 m).

Horizontal mass flux above the surface varied as a function of height (Figure 2). For all sampling periods, the highest mass flux was observed at a height of 0.1 m, which was the lowest placement height of BSNE collectors. The mass flux profile is well represented by the power-law equation (Eq.1) as a good relationship was found between height and horizontal mass flux within 2 (R^2^ > 0.9) and 10 m (R^2^ > 0.77) of the surface. Mass flux was small at a height of 2 m (3–82 g m^−1^) and 10 m (0.04–45 g m^−1^) across all events (Figure 2). We are not aware of previous studies that have documented sediment mass flux at a height of 10 m because much of the sediment is transported below a height of 2 m^11, 21^. Aggregate or primary particle size of aeolian sand at the desert–oasis ecotone site was finer than the aggregate size distribution of agricultural soils in the Tarim Basin. For example, the geometric mean diameter of the aggregate size distribution was 0.254 mm at the desert–oasis ecotone site^22^ and 3.600 and 5.310 mm for a cotton field and red date orchard near Alaer City^35^. Thus, aeolian sand may be more erodible than agricultural soils in the region.

For all sampling periods, sediment trapped by creep or BSNE collectors was characterized by a diameter of <0.84 mm. The aggregate and primary particle size distributions of the Aeolian sand at our experiment site indicated no aggregates or particles >0.84 mm^22^. *Pi et al*.^22^ reported the percentage of aggregates <0.075, 0.075–0.1, 0.1–0.25, 0.25–0.5, and 0.5–0.84 mm in diameter were respectively 1, 16, 38, 45, and 0%. Likewise, the percentage of primary particles <0.07, 0.07–0.1, 0.1–0.25, 0.25–0.5, and 0.5–0.84 mm in diameter were respectively 7, 18, 66, 9, and 0%. The high proportion of fine particles constituting the aeolian sand and trapped by BSNE collectors indicated potential for saltation and suspension to dominate the erosion process.

**Figure 3:**
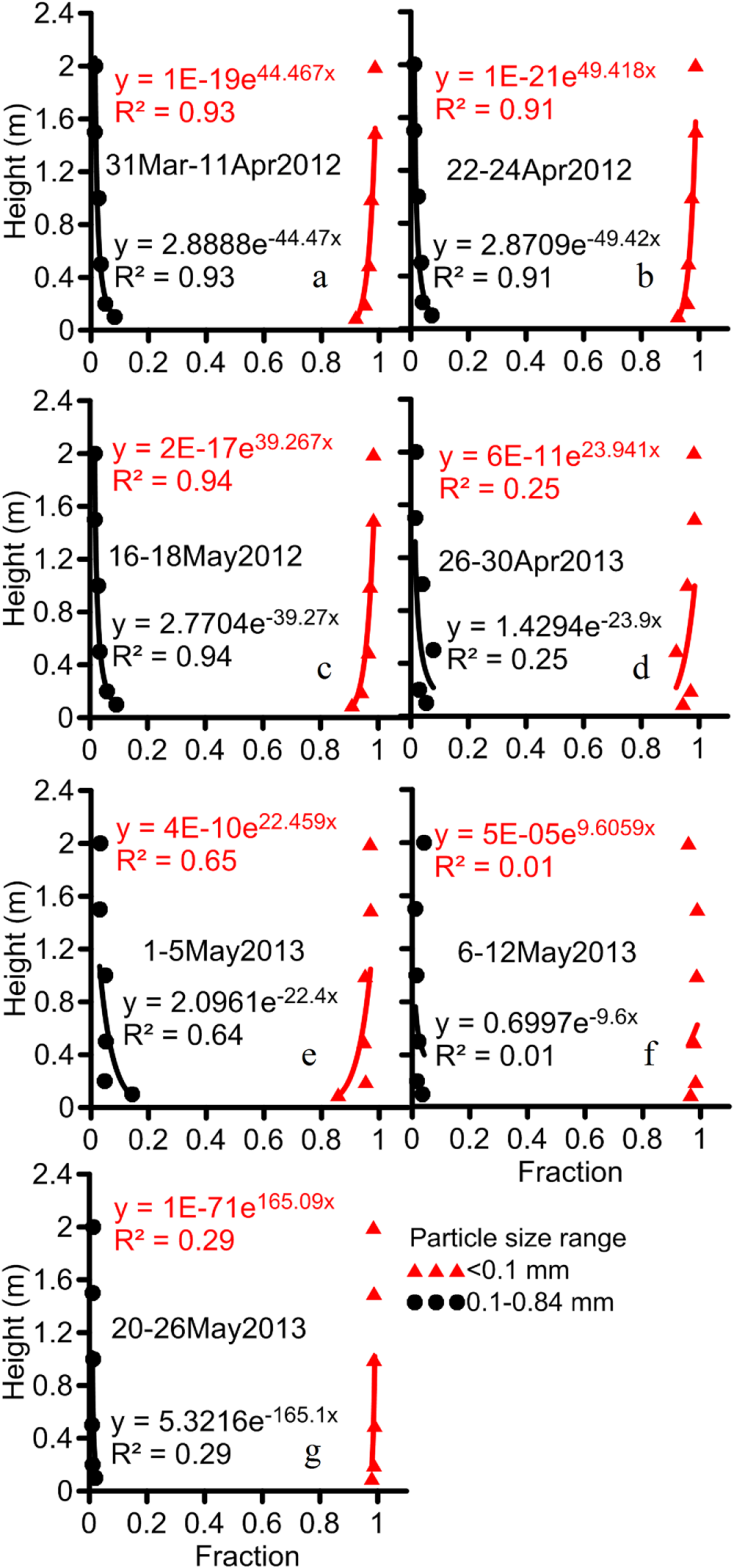
Size distribution of sediment trapped by BSNE collectors during 7 high wind events. Sediment was trapped by BSNE collectors positioned at six heights (0.025–2 m).

The size distribution of sediment trapped by BSNE collectors to a height of 2 m is reported in Figure 3. Saltation-size particles comprised from 2.1 to 14.3% of the sediment trapped at 0.1 m height and 1.2 to 4.2% of sediment trapped at 2 m height across all sample periods. Based upon the size composition of sediment at 2 m, all sediment trapped by the BSNE at heights >2 m was estimated to be <0.1 mm. A exponential function describing the size distribution with height (Figure 3) indicated the mass fraction of particles <0.1 mm increased with the height whereas the mass fraction of particles 0.1–0.84 mm decreased with the height across all sample periods. Based upon these functions, the height at which the mass fraction of particles <0.1 mm attained a value of 97.8% was 2 m and the height at which the mass fraction of particles 0.1–0.84 mm attained a value of 7.1% was 0.1 m averaged across all sample periods. Suspension-size particles have been designated as particles with diameters <0.1 mm^36–38^. Sediment loss by suspension ranged from 100 to 2437 g m^−2^, or 67.4 to 84.8% of the total sediment loss across all high wind events. We recognize the efficiency of the BSNE is influenced by the geometric mean diameter of the suspended sediment with the efficiency of the BSNE being lower for finer particles^27^. As some of the collected sediment had geometric diameters less than 0.1 mm, the measured sediment loss due to the possibility of low efficiency of BSNE may underestimate the real sediment loss.

## Conclusion

Sediment loss was assessed from an aeolian sand a desert–oasis ecotone in Northwesten Tarim Basin during 2012 and 2013. The highest loss observed from an aeolian sand in this study was 2925 g m^−2^. Wind played a dominant role in sediment loss at the desert–oasis ecotone site. The variation in horizontal mass flux within 10 m of the eroding surface was well represented by a power function. Little sediment (0.04~45 g m^−1^) was captured by the BSNE collectors at a height of 10 m. Our results justify the importance of implementing practices to control wind erosion and thus guarantee ecological security of the desert-oasis ecotone.
